# N-acetylglucosamine Regulates Virulence Properties in Microbial Pathogens

**DOI:** 10.1371/journal.ppat.1004947

**Published:** 2015-07-30

**Authors:** Shamoon Naseem, James B. Konopka

**Affiliations:** Department of Molecular Genetics and Microbiology, Stony Brook University, Stony Brook, New York, United States of America; Duke University Medical Center, UNITED STATES

## Introduction

There is growing evidence that the sugar N-acetylglucosamine (GlcNAc) plays diverse roles in cell signaling pathways that impact the virulence properties of microbes and host cells. GlcNAc is already well known as a ubiquitous structural component at the cell surface that forms part of bacterial cell wall peptidoglycan, cell wall chitin in fungi and parasites, and extracellular matrix glycosaminoglycans of animal cells. Chitin and peptidoglycan have been previously linked to cell signaling as they can stimulate responses in plant and animal host cells [1–3]. Recent studies now indicate that GlcNAc released from these polymers can also activate cell signaling via several different mechanisms [4–6]. The role of these new GlcNAc signaling pathways in the regulation of virulence factors will be the focus of this review.

## GlcNAc Induces Morphogenesis and Virulence Pathways in Fungi

**Fig 1:**
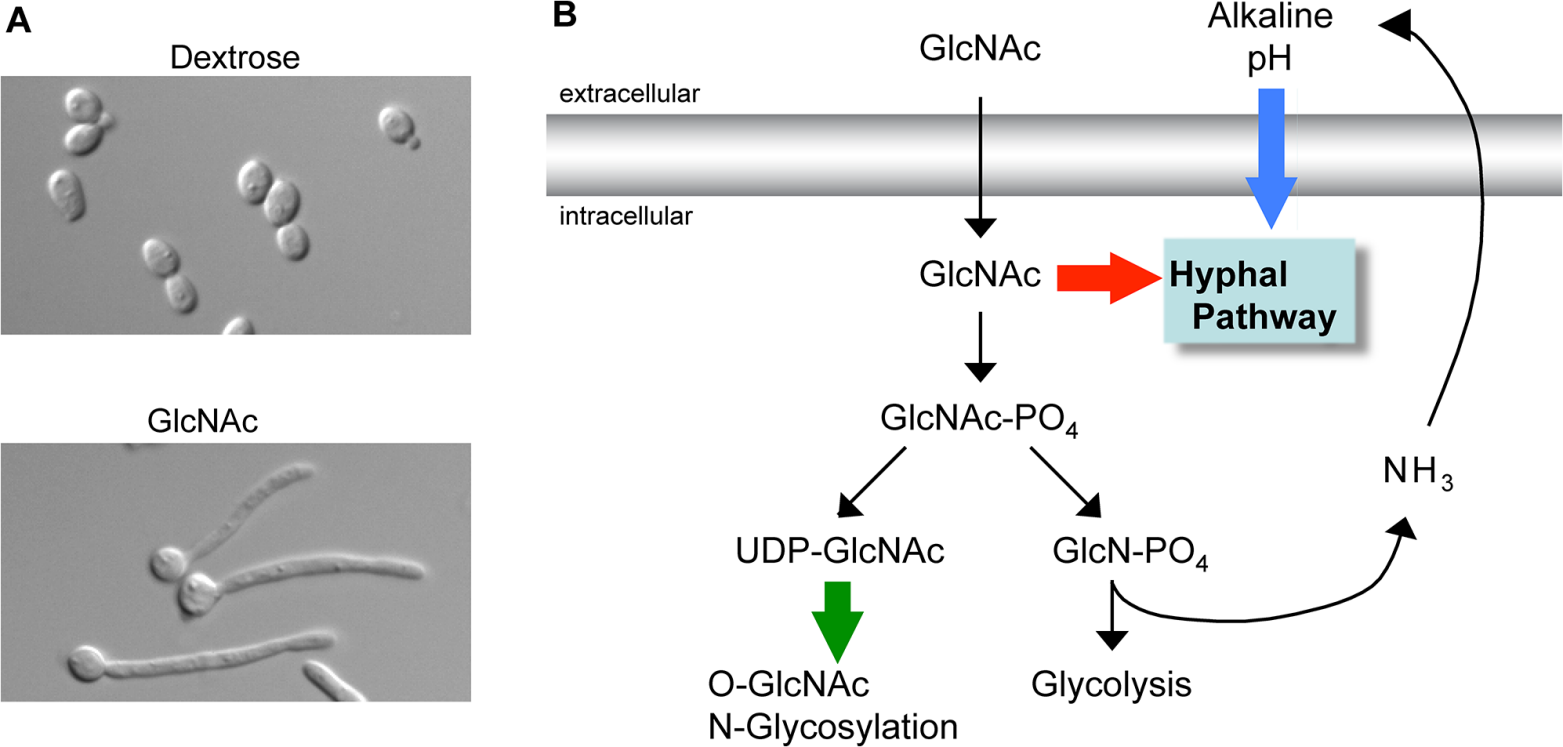
GlcNAc signaling pathways. (A) *C*. *albicans* grown in dextrose form budding cells (top) whereas growth in GlcNAc induces them to switch to the filamentous hyphal form (bottom). (B) Summary of three types of GlcNAc-regulated pathways. GlcNAc itself can transduce a signal to induce hyphal growth in *C*. *albicans* (red arrow). Catabolism of GlcNAc releases excess ammonia whose export alkalinizes the extracellular pH and can synergize with GlcNAc to induce hyphal growth and gene expression (blue arrow). In mammals and some microbes conversion of GlcNAc to the building block UDP-GlcNAc promotes changes in O-GlcNAc modification of intracellular proteins and N-linked glycosylation of cell surface proteins (green arrow).

GlcNAc first attracted attention as a signaling molecule for fungi over 40 years ago, when it was discovered to induce a remarkable switch from budding to hyphal growth in the human pathogen *Candida albicans* (Fig 1A) [7]. GlcNAc was subsequently shown to induce filamentous growth in a diverse group of fungi [5]. Switching to filamentous hyphal morphology contributes to invasive growth of *C*. *albicans* in the host and influences the interaction with leukocytes [8]. GlcNAc also stimulates the expression of virulence genes, such as the adhesins that promote adherence to host cells and biofilm formation [5,8]. Although it is not clear whether GlcNAc plays a role in systemic candidiasis, it has been implicated in commensal growth in the mucosa of the GI tract [9]. Consistent with this, GlcNAc promotes an epigenetic switch in morphology from the “White Phase” to the “Opaque Phase,” which is better adapted to mucosal growth [10].

Identification of a GlcNAc transporter (Ngt1) in the *C*. *albicans* plasma membrane helped to resolve earlier controversies as to whether GlcNAc had to be imported into the cell to induce signaling [11]. An *ngt1Δ* mutant was defective in inducing hyphae, indicating that intracellular GlcNAc activates signaling. Since Ngt1 was the first eukaryotic GlcNAc transporter to be identified, its discovery has also helped to define the role of GlcNAc transport in other species. An interesting example of this is that Ngt1 orthologs were shown to mediate the ability of GlcNAc to induce hyphal growth in the dimorphic fungal pathogen *Histoplasma capsulatum* [12].

The ability of intracellular GlcNAc to transduce a signal raised the question of whether it had to be metabolized to induce signaling. Analysis of a *C*. *albicans* mutant lacking all three enzymes needed for GlcNAc catabolism (*hxk1Δ nag1Δ dac1Δ*) showed that the breakdown of this sugar was not needed for it to promote hyphal growth [13]. Furthermore, analysis of this mutant also indicated that GlcNAc did not have to be converted to the important building block UDP-GlcNAc. The *hxk1Δ* mutation blocks conversion of GlcNAc to GlcNAc-6-PO_4_, which is required for it to be subsequently processed into UDP-GlcNAc. These results indicate *C*. *albicans* uses a novel GlcNAc pathway (red arrow in Fig 1B) that is distinct from the major known signaling pathway in mammalian cells that requires conversion of GlcNAc into UDP-GlcNAc for use in O-GlcNAc modification of intracellular proteins (green arrow) [14]. The search for components in *C*. *albicans* that transduce the GlcNAc signal indicates that multiple pathways are activated. For example, the cAMP pathway is needed for GlcNAc to induce hyphal morphogenesis and virulence genes, but is not needed to induce the genes needed to catabolize GlcNAc [15].

## Catabolism of GlcNAc Raises the Ambient pH: Synergy between GlcNAc and pH

Although GlcNAc catabolism is not required to induce hyphae, it can indirectly stimulate responses in *C*. *albicans* by raising the pH of the extracellular medium (blue arrow in Fig 1B) [16]. In contrast to acidification of the environment that occurs for cells grown in glucose, growth in GlcNAc raises the pH since cells export excess nitrogen as ammonia [17]. Studies with a mutant that lacks the GlcNAc metabolic genes (*hxk1Δ nag1Δ dac1Δ*) revealed an interesting synergy between GlcNAc and pH [16] as the mutant cells were able to induce hyphal morphology without the induction of hyphal-specific genes at low pH. However, the mutant cells could induce hyphal-specific genes when buffered to a higher pH (>5) that mimicked the effects of GlcNAc catabolism. Although alkaline pH can induce hyphal responses [8], the observed effects occurred at pH levels that were well below the levels required to induce hyphae, indicating that there is synergy between these pathways. These results are significant because they indicate that GlcNAc can stimulate hyphal morphogenesis independently of the induction of hyphal-specific genes, which had been linked to promoting the transition to filamentous growth [8]. This type of synergy between GlcNAc and pH likely occurs with other species, as cells from bacteria to humans export excess nitrogen as ammonia [18]. Thus, future studies must take care to distinguish between a direct role of GlcNAc in cell signaling and an indirect effect on the ambient pH.

## GlcNAc Regulation of Virulence Factors in Bacteria

An important source of GlcNAc for cell signaling in many environments is due to release of this sugar during bacterial growth due to remodeling of cell wall peptidoglycan, which consists of alternating GlcNAc and N-acetylmuramic acid residues [19]. Approximately 50% of the sidewall peptidoglycan is broken down during each generation to accommodate the stepwise enlargement of the cell wall [19]. The presence of exogenous GlcNAc can therefore signal that cells nearby are dividing. One metabolic decision regulated by exogenous GlcNAc is to determine whether cells synthesize new GlcNAc, recycle exogenous GlcNAc back into peptidoglycan, or catabolize it for nutrition. *Escherichia coli* has streamlined this decision by placing the genes needed for GlcNAc synthesis and catabolism on opposite sides of a divergent operon that is regulated by the NagC transcription factor that responds to GlcNAc-6-PO_4_ [20]. Proper regulation of GlcNAc metabolism genes is significant, as it is important for colonization of the host by *E*. *coli* [21] and *Vibrio cholera* [22], and for production of virulence factors and biofilms by the cariogenic bacterium *Streptococcus mutans* [23].

GlcNAc has diverse effects in different bacteria by up-regulating or down-regulating virulence factors. In soil bacteria, it stimulates antibiotic production [24]. In polymicrobial infections, GlcNAc released from Gram-positive bacteria makes the infection more severe by stimulating *Pseudomonas aeruginosa* to produce toxins and virulence factors [6,25]. In contrast, GlcNAc down-regulates two extracellular adhesion factors in *E*. *coli*. GlcNAc inhibits production of type 1 fimbrial adhesins that promote urinary tract infections by mediating attachment to host cells [4]. GlcNAc also diminishes the production of the extracellular Curli fibers that play a role in biofilm formation, adhesion, and the internalization of *E*. *coli* by epithelial cells [26]. It has been suggested that rising GlcNAc levels during inflammation could signal to bacteria that host defenses are activated [4]. Inhibiting the expression of fimbriae and Curli fibers would therefore have two advantages for the bacteria: it would decrease the levels of these pro-inflammatory surface structures and the decreased levels of these adhesins would promote dissemination within the host. In this regard, it is interesting that many bacteria adhere via biofilms formed with extracellular poly-β,1–6 GlcNAc (distinct from β,1–4 linked chitin). Degradation of poly-β,1–6 GlcNAc disperses cells from biofilms and would also likely activate GlcNAc signaling that could affect adhesin production to further promote dissemination.

GlcNAc has additional roles in bacterial pathogenesis that depend on its metabolism and conversion to UDP-GlcNAc. For example, O-GlcNAc modification of proteins regulates cell motility in the pathogen *Listeria monocytogenes* [27]. In addition, exported toxins in other bacteria promote an unusual O-GlcNAc modification on arginine residues in cell death receptors and tyrosine residues in Rho that inactivates these host functions [28,29].

## How Do Cells Distinguish Exogenous Versus Endogenous GlcNAc?

A key question is how do cells sense exogenous GlcNAc when they actively synthesize high levels of this sugar to create UDP-GlcNAc, a building block for glycosylation, GPI anchors, and the cell wall. Fungi and bacteria appear to distinguish exogenous GlcNAc because its phosphorylation status is distinct from endogenously synthesized GlcNAc [5]. For example, fungi take up unmodified GlcNAc, whereas they synthesize a phosphorylated form (GlcNAc-6-PO_4_) [30]. A variation of this occurs in bacteria that take up GlcNAc using a phosphotransferase system that converts it to GlcNAc-6-PO_4_, a form that is not synthesized in bacteria. Bacteria differ from fungi in that they avoid making GlcNAc-6-PO_4_ because the precursor sugar, glucosamine-6-PO_4_, is converted directly to GlcNAc-1-PO_4_, which is then further modified to create UDP-GlcNAc [30].

## GlcNAc Signaling in Mammalian Cells

GlcNAc is known to induce responses in mammalian cells following its conversion to UDP-GlcNAc (green arrow in Fig 1B). Elevated UDP-GlcNAc increases O-GlcNAc modification of proteins and also increases N-GlcNAc branching on cell surface proteins, which changes cell signaling properties by altering the stability of receptors on the cell surface [14,31]. It is not clear whether GlcNAc itself can induce signaling in mammals. However, it is noteworthy that after infection with the fungus *Cryptococcus neoformans*, Th2 cell induction depended on cleavage of chitin by the mammalian chitinase, chitotriosidase, indicating that chitin fragments and perhaps GlcNAc are involved [32]. Although GlcNAc has also been reported to inhibit Th1 and Th17 cells, which play key roles in antifungal defense [33], further studies will be needed to determine how GlcNAc influences the immune system.

## Concluding Comments

Emerging data indicate that the ubiquitous sugar GlcNAc is sensed by a broad range of organisms as a way to detect growth of neighboring cells or pathogenic attack. The ability of fungal and bacterial pathogens to regulate virulence functions in response to GlcNAc suggests that parasites will too, a possibility supported by the important role for GlcNAc metabolism in *Leishmania* [34]. The widespread presence of GlcNAc also suggests that is well suited to mediate interspecies communication with the host or between microorganisms to promote either symbiotic relationships or pathogenic interactions. In this way, GlcNAc is similar to many different chemical messengers, including quorum sensing factors, that are also used to communicate both intra- and interspecies [35]. Thus, it will be important to define the roles for GlcNAc signaling in complex environments, such as the human gut or in polymicrobial infections that contain a diverse array of bacteria, fungi, and human cells [36].
